# Mutational Analysis of *Trypanosoma brucei* RNA Editing Ligase Reveals Regions Critical for Interaction with KREPA2

**DOI:** 10.1371/journal.pone.0120844

**Published:** 2015-03-19

**Authors:** Vaibhav Mehta, Rajashree Sen, Houtan Moshiri, Reza Salavati

**Affiliations:** 1 Department of Biochemistry, McGill University, McIntyre Medical Building, 3655 Promenade Sir William Osler, Montreal, Quebec, H3G1Y6, Canada; 2 Institute of Parasitology, McGill University, 21111 Lakeshore Road, Ste. Anne de Bellevue, Montreal, Quebec, H9X3V9, Canada; 3 McGill Centre for Bioinformatics, McGill University, Duff Medical Building, 3775 University Street, Montreal, Quebec, H3A2B4, Canada; University of Texas Medical School at Houston, UNITED STATES

## Abstract

The *Trypanosoma brucei* parasite causes the vector-borne disease African sleeping sickness. Mitochondrial mRNAs of *T*. *brucei* undergo posttranscriptional RNA editing to make mature, functional mRNAs. The final step of this process is catalyzed by the essential ligase, *T*. *brucei* RNA Editing Ligase 1 (*Tb*REL1) and the closely related *T*. *brucei* RNA Editing Ligase 2 (*Tb*REL2). While other ligases such as T7 DNA ligase have both a catalytic and an oligonucleotide/oligosaccharide-binding (OB)-fold domain, *T*. *brucei* RNA editing ligases contain only the catalytic domain. The OB-fold domain, which is required for interaction with the substrate RNA, is provided in *trans* by KREPA2 (for *Tb*REL1) and KREPA1 (for *Tb*REL2). KREPA2 enhancement of *Tb*REL1 ligase activity is presumed to occur via an OB-fold-mediated increase in substrate specificity and catalysis. We characterized the interaction between *Tb*REL1 and KREPA2 *in vitro* using full-length, truncated, and point-mutated ligases. As previously shown, our data indicate strong, specific stimulation of *Tb*REL1 catalytic activity by KREPA2. We narrowed the region of contact to the final 59 C-terminal residues of *Tb*REL1. Specifically, the *Tb*REL1 C-terminal KWKE (441–444) sequence appear to coordinate the KREPA2-mediated enhancement of *Tb*REL1 activities. N-terminal residues F206, T264 and Y275 are crucial for the overall activity of *Tb*REL1, particularly for F206, a mutation of this residue also disrupts KREPA2 interaction. Thus, we have identified the critical *Tb*REL1 regions and amino acids that mediate the KREPA2 interaction.

## Introduction

The mitochondrial DNA of trypanosomes contains a structure called the kinetoplast, comprising a large network of concatenated DNA organized into two circular structures [1, 2]. The larger of the two are “maxicircles,” which are present at 50–100 copies per cell and encode mitochondrial proteins, including those involved in the mitochondrial respiratory chain and oxidative phosphorylation [3]. However, kinetoplast mRNA is first transcribed in an unedited or encrypted form that needs to undergo posttranscriptional processing or “RNA editing” [4] by uridylate (U) insertion or less frequently by uridylate deletion, as specified by the sequence of guide RNAs (gRNAs), to become fully functional RNA. The most extensive editing is seen in *Trypanosoma brucei*, where approximately 50% of mitochondrial mRNA sequences undergoes remodeling by editing and the type of mRNA edited reflects the varying forms of energy metabolism [5].

RNA editing is catalyzed by a multiprotein complex called the “editosome,” containing at least 20 different proteins (see reviews [6, 7]). The editing of each site involves an “enzyme-cascade” mechanism. In the first enzymatic step, an editing site-specific endonuclease recognizes the mRNA/gRNA anchor duplex and cleaves the mRNA 3′ to the first unpaired nucleotide. Next, U residues are either added to the 5′ cleavage fragment by a terminal uridylyl transferase (TUTase) in insertion editing or removed by a 3′ U-specific exonuclease in deletion editing. Finally, the modified mRNA is rejoined by an RNA ligase. This final step is catalyzed by either *T*. *brucei* RNA Editing Ligase 1 (*Tb*REL1) or the closely related *T*. *brucei* RNA Editing Ligase 2 (*Tb*REL2).

The ligation mechanism of *Tb*REL1 is similar to that of DNA ligases, although their structures are different. The ligation reaction follows three steps: (i) an adenylylation step, in which the conserved catalytic lysine attacks a phosphate of ATP and releases pyrophosphate (at this step, an enzyme-AMP intermediate is formed through a phosphoamide linkage); (ii) a deadenylylation step, in which *Tb*REL1-AMP recognizes double-stranded nicked mRNA/gRNA and transfers its bound AMP to the 5′ phosphate of the RNA molecule to form adenylylated RNA with a 5′-5′-phosphoanhydride bond; and (iii) a ligation step, in which the free 3′ hydroxyl of the 5′ fragment attacks the phosphoanhydride bond of the adenylylated 3′ RNA fragment at the nick site, leading to the formation of a phosphodiester bond and the release of AMP [8].

Parallel ligase functions have been suggested for the two ligases, with *Tb*REL1 specifically catalyzing RNA deletion editing and *Tb*REL2 specifically catalyzing RNA insertion editing [9]. Interestingly, although 90% of trypanosomal editing involves U insertion, RNAi-mediated *Tb*REL2 silencing does not change U insertion RNA editing or cell survival rates of the different *T*. *brucei* life stages. This suggests a nonessential role for *Tb*REL2 in RNA editing [10]. However, a null mutant of *Tb*REL2 has not yet been reported, and it is possible that very small amounts of *Tb*REL2 remaining after gene knock down can adequately support editing. In contrast, *Tb*REL1 has been firmly shown to be an integral part of the editosome complex and essential for RNA editing in *T*. *brucei* [11]. While all other DNA ligases and mRNA capping enzymes have an N-terminal adenylylation domain and a C-terminal oligonucleotide/oligosaccharide-binding (OB) fold, *Tb*REL1 and *Tb*REL2 have only the N-terminal adenylylation domain and contain a completely divergent C-terminal domain [9]. T4 RNA ligase 2 is the closest ligase to the RNA editing ligases [12]. T4 RNA ligase 2 and RNA editing ligases contain a conserved N-terminal adenylyltransferase domain, with a unique C-terminal domain that has no sequence or structure similarity to known OB-fold domains conserved in all other ligases [12, 13].

In the editosome, *Tb*REL1 and *Tb*REL2 associate with their interacting partners KREPA2 and KREPA1, respectively, which provide the OB folds in *trans* to the ligases. In fact, *in vitro* studies have shown that KREPA2 stimulates *Tb*REL1-mediated ligation and KREPA1 stimulates *Tb*REL2-mediated ligation [9]. Along with the OB fold, the interacting partners also contain two C2H2 zinc-finger motifs in their N termini [9, 14]. While OB folds function in nucleic acid recognition [15], zinc fingers are implicated in editosome complex association, presumably through mediating protein-protein or protein-RNA binding [16, 17]. KREPA2 is essential for assembly of the editosome complex: RNAi-mediated KREPA2 depletion disassembles the complex and results in a loss of *Tb*REL1 [18]. Moreover, KREPA2 depletion severely impairs the growth of insect-stage trypanosomes. Similarly, RNAi-mediated KREPA1 depletion inhibits U editing and growth of both the bloodstream and insect stages of *T*. *brucei* [10]. *Tb*REL1 is a candidate anti-trypanosome drug target. As both *Tb*REL1 and its interacting partner KREPA2 are important proteins in *T*. *brucei* growth, further investigations into their mode of interaction are warranted.

In this study, we identified functionally important *Tb*REL1 regions that mediate its ligase activity by measuring the effect of adding KREPA2 to wild-type and truncated *Tb*REL1. We also identified the functionally important amino acids in *Tb*REL1 that coordinate its interaction with KREPA2.

## Experimental Procedures

### Cloning of full-length TbREL1 and KREPA2

The sequence corresponding to *Tb*REL1 open reading frame (ORF; minus the mitochondrial import signal) was excised from the original pSG1-*Tb*REL1 construct [14] and cloned into a pET-30a vector between restriction sites KpnI and XhoI. The recombinant protein expressing from this plasmid contains an N-terminal 6×His tag. KREPA2 DNA corresponding to the KREPA2 ORF was cloned into the pSG vector between the EcoRI and BamHI restriction sites, as previously described [14].

### Cloning of TbREL1 truncations and point mutations

Three *Tb*REL1 truncations, *Tb*REL1-N-terminal, *Tb*REL1-R372, and *Tb*REL1-DALKD, were made by creating KpnI/XhoI DNA fragments by PCR using full-length *Tb*REL1 as a template and inserting the cloned fragments into a KpnI/XhoI-digested pET-30a vector. A total of 20 individual point mutations were made in *Tb*REL1 by site-directed mutagenesis of *Tb*REL1-pet30a. All truncations and point mutations were prepared by GenScript Corporation (Piscataway, NJ).

### In vitro protein expression

**Fig 1:**
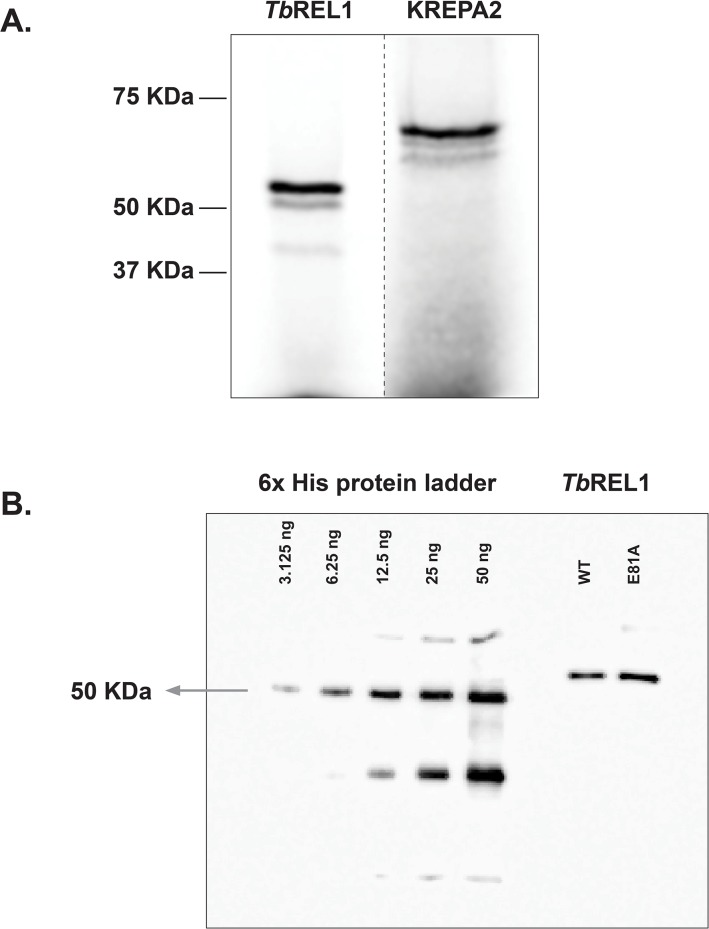
Expression of *Tb*REL1 and KREPA2. (A) Both proteins were *in vitro* transcribed/translated in the presence of [^35^S]-methionine and separated by 10% SDS-PAGE. *Tb*REL1 (52 kDa) and KREPA2 (63 kDa) migrated at the expected size. (B) Western blot analysis of His-tagged *Tb*REL1. The blot shows 0.3125, 0.625, 1.25, 2.5, and 5 μL of a 6x His protein ladder of known amounts (3.125, 6.25, 12.5, 25 and 50 ng corresponding to the 50 KDa protein) along with 2 μL of purified *Tb*REL1 WT and mutation E81A.

Recombinant proteins were synthesized *in vitro* using a Reticulocyte lysate based cell-free coupled transcription and translation system (TNT; L1170, Promega). All *Tb*REL1 plasmids expressed the protein with an N-terminal 6×His tag and KREPA2 protein was expressed untagged. The efficiency of full-length protein expression, containing [^35^S] methionine (NEG709A500UC, Perkin Elmer) was analyzed by SDS-PAGE and visualized by PhosphorImager (Fig. 1A). Both proteins migrated at the expected sizes: 52 kDa for *Tb*REL1 and 63 kDa for KREPA2. Radiolabeled proteins were analyzed using the Quantity One software (Bio-Rad). This software was also used for quantification of data in this paper with the aid of its volume measurement tools. Non-radiolabeled proteins were examined by Western blotting using an anti-6x His tag antibody (631212, Clontech) and visualized by VersaDoc (Bio-Rad). Proteins in these gels were quantified with the aid of 6xHis protein ladder (34705, Qiagen) using the Quantity One software (Fig. 1B). The 6xHis Protein ladder is loaded in varying amounts for quantitation, i.e. 0.3125 μL, 0.625 μL, 1.25 μL, 2.5 μL and 5 μL; when 2.5 μL of the ladder is loaded, the 50 KDa protein band corresponds to 25 ng, thereby, correlating to 3.125 ng, 6.25 ng, 12.5 ng, 25 ng and 50 ng of the 50 KDa protein in these lanes. A standard curve is then plotted using these values and the intensities of the 50 KDa protein in the respective lanes. Finally, the concentrations of *Tb*REL1 proteins are measured using the equation obtained (Fig. 1B, S1 Fig.).

### Precipitation and elution

*Tb*REL1 proteins expressed using the TNT system, were precipitated using magnetic beads for His-tag protein purification (Dynabeads 10103D, Life Technologies). For a 50 μL TNT reaction, 10 μL of the magnetic beads were used. The beads were first washed once in ddH_2_O and then twice in blocking solution (25 mM NaPO_4_ [pH 8.0], 200 mM NaCl, 1% BSA and 0.01% Tween 20). They were then resuspended in 500 μL blocking solution and incubated on a tube rotator for 1 h. The beads were then washed twice with binding buffer (50 mM NaPO_4_ [pH 8.0], 300 mM NaCl and 0.01% Tween 20), resuspended in 500 μL of the same and added to the TNT reaction. If *Tb*REL1 samples were to be prepared with KREPA2, equal volume of KREPA2 TNT mixtures were added to the respective *Tb*REL1 TNT mixtures just before adding the magnetic beads to it. The mixture was incubated on a tube rotator for 20–30 min at 4°C. The beads were subsequently washed thrice in binding buffer. For non-radiolabeled protein samples used in testing *Tb*REL1 activity, the beads were then resuspended in 10–20 μL of 1×HHEG (25 mM Hepes [pH 7.9], 50 mM KCl, 10 mM Mg (OAc)_2_, 1 mM EDTA and 20% glycerol) and flash frozen in liquid nitrogen to be stored at-80°C for future use. For the [^35^S]-methionine labeled based co-precipitation experiment, the beads were resuspended in 20 μL of SDS-PAGE loading dye instead, and run on a 10% SDS-PAGE gel and visualized by PhosphorImager.

[^35^S]-methionine-labeled *Tb*REL1 proteins to be analyzed on Native-PAGE were eluted right after three washes using 500 μL of an imidazole containing buffer (50 mM NaPO_4_ [pH 8.0], 300 mM NaCl, 0.01% Tween 20 and 300 mM Imidazole) for 20–30 min on a tube rotator at RT. The eluates were then concentrated using 10 KDa MWCO centrifugal filter concentrators (UFC501024, Millipore) to a usable volume, approximately 30 μL. To this concentrated solution, 450 μL of a Native buffer (20 mM Tris-HCl [pH 7.5], 50 mM KCl) was added and concentrated again to 30 μL. This step was repeated twice to ensure the buffer exchange. To store the samples, glycerol was added to a final concentration of 20%, flash frozen and stored at −80°C.

### Native PAGE

[^35^S]-methionine labeled *Tb*REL1 protein preparations were resolved on 4–16% Novex Bis-Tris native gels (BN1004BOX, Life Technologies). Samples were prepared using the sample preparation kit (BN2008, Life Technologies). The samples were run on gels as soon as they were prepared at 150V for 45–60 min and visualized using PhosphorImager.

### Adenylylation reactions

Adenylylation reactions were carried out using 0.3 pmol of *Tb*REL1 proteins (wild-type and mutants) with and without KREPA2. The reactions were incubated with 20 μCi [α-^32^P]ATP in a buffer containing 25 mM Tris-HCl [pH 8.0], 10 mM Mg(OAc)_2_, 0.5 mM DTT, 1% BSA and 10% DMSO for 15 min at RT (final volume is 30 μL) (protocol adapted from [19, 20]). Adenlylation was stopped by the addition of SDS-PAGE loading dye. Proteins were resolved on a denaturing SDS-PAGE gel and detected using a PhosphorImager.

### Pre-cleaved RNA ligation assay

RNA fragments used in the pre-cleaved RNA editing ligation assay were (a) 5′ RNA fragment **5′lig**, 5′- GGAAAGUUGUGACUGA-3′, (b) 3′ RNA fragment **3**′**lig**, 5′- pUGAGUCCGUGAGGACGAAACAAUAGAUCAAAUGUp-3′ (c) guide RNA **glig**, 5′- GUUUCGUCCUCACGGACUCAUCAGUCACAACUUUCC-3′ and (d) guide RNA competitor (DNA) **gligC**, 5’- GGAAAGTTGTGACTGATGAGTCCGTGAGGACGAAAC-3’. The following oligos, 3’lig, glig and gligC were synthesized and HPLC purified by Integrated DNA Technologies (IDT; Coralville, IA). 5’lig was prepared from *in vitro* transcription of the PCR product of the DNA dimer, **5’ligDNAtemp**, 5’- CGGCGGAATTCTGTAATACGACTCACTATAGGAAAGTTGTGACTGA-3’ by the primers **5’ligFwd** 5’- CGGCGGAATTCTGTAATACGACTCAC-3’ and **5’ligRev**, 5’- TCAGTCACAACTTTCCTATAG-3’ (all oligos were synthesized by IDT). The T7 RiboMAX kit (P1320, Promega) was used for transcribing the 5’lig RNA *in vitro*. The RNAs were subsequently resolved on a 15% denaturing polyacrylamide gel (7 M urea), gel eluted and ethanol precipitated for purification. The 5’lig RNA oligo was then capped with [α-32P] GTP (BLU006H250UC, Perkin Elmer) using the ScriptCap m7G Capping System (Cellscript) and PAGE purified as described above.

For ligation reactions, [α-^32^P]-GTP capped 5′lig (0.25 μM) and 3′lig (1 μM) fragments were annealed to glig (0.5 μM) at 70°C for 2 min and cooled to RT for 15 min. Annealed RNA substrates were then added to the editing reaction buffer containing 1×HHE (25 mM Hepes [pH 7.9], 50 mM KCl, 10 mM Mg (OAc)_2_, and 1 mM EDTA), 1 mM ATP, 83 ng/mL total torula yeast RNA, and 5 mM CaCl_2_. This RNA buffer master mix was then added to 0.3 pmol *Tb*REL1 proteins, with and without KREPA2, and the reaction was initiated at 28°C (final volume is 30 μL) (protocol adapted from [21]. RNA ligation reactions were stopped after 3 h by adding 2 μL of stop buffer (2.5% SDS and 130 mM EDTA). Guide competitor DNA, gligC was added to each reaction to a final concentration of 10 μM. RNA were extracted using phenol:chloroform:isoamyl alcohol (25:24:1, pH 6.7) and separated on a 15% polyacrylamide gel containing 7 M urea for 2 h at 40 W and visualized using a PhosphorImager.

## Results

### KREPA2 significantly stimulates TbREL1 adenylylation and ligation

**Fig 2:**
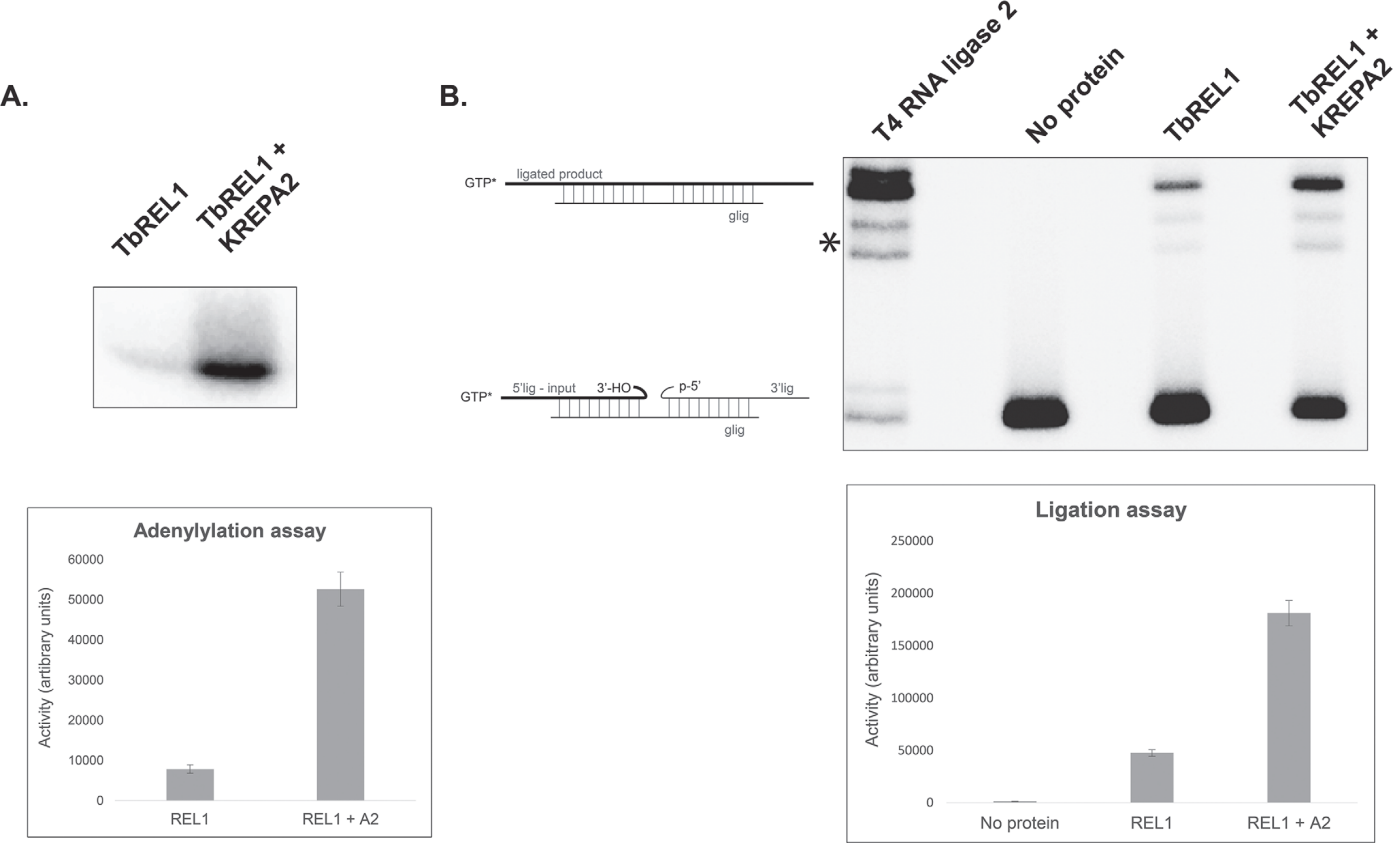
KREPA2 stimulates *Tb*REL1 activity. (A) Stimulatory effect of KREPA2 on *Tb*REL1 adenylylation. A graph representing the adenylylation enhancement (6.7 fold) achieved by adding KREPA2 is seen below. Here, the X-axis represents the adenylylation activity (arbitrary values obtained from Quantity One volume measurement tool). The error bars represent standard deviation between triplicate samples. (B) Stimulatory effect of KREPA2 on *Tb*REL1-mediated ligation. Two minor bands (single star) are a result of nonspecific ligation of input degradation products. The illustration shows the input 5′ [α-^32^P]-GTP-capped RNA fragment along with the unlabeled 3′ fragment and gRNA, and the [α-^32^P]-GTP-capped ligated product. A graph representing the stimulation of ligation achieved by adding KREPA2 (3.6 fold) is seen below. Here, the X-axis represents the ligation activity (arbitrary values obtained from Quantity One volume measurement tool). The error bars represent standard deviation between triplicate samples.

To investigate the effect of KREPA2 on *Tb*REL1 activity, the adenylylation and ligation efficiencies of the wild-type *Tb*REL1 was tested *in vitro* in the presence and absence of equimolar amounts of KREPA2. In both assays, the enzymatic activity of *Tb*REL1 was considerably enhanced in the presence of KREPA2 (Fig. 2). Measurement of the enhancement achieved in presence of KREPA2 showed an increase of 6.7 fold in adenylylation and 3.6 fold in ligation activities of *Tb*REL1 (Fig. 2). Conclusively, KREPA2 seems to play an important role in regulating the catalytic activities of *Tb*REL1.

### Expression of TbREL1 truncations

**Fig 3:**
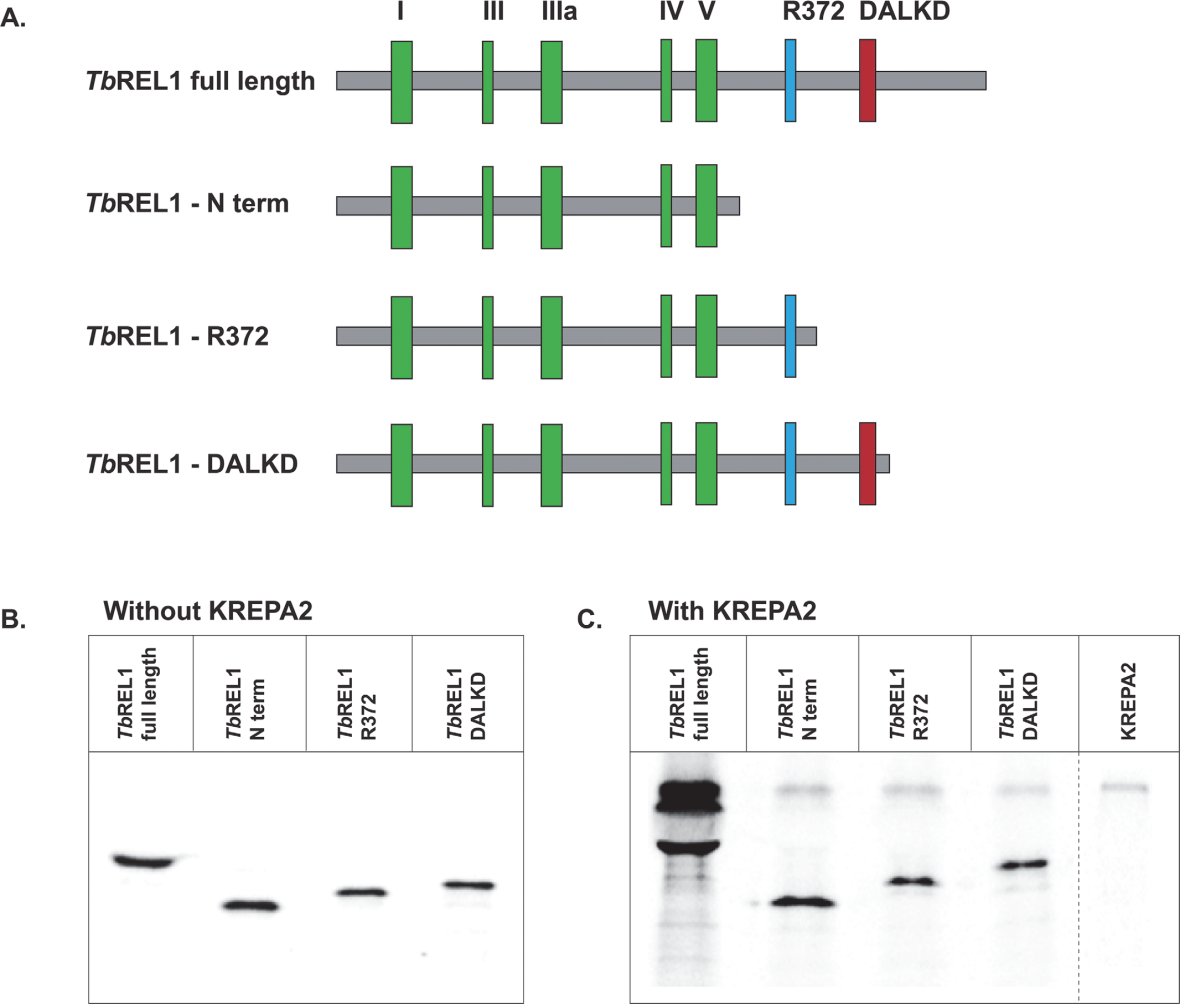
Three *Tb*REL1 truncation mutants. A) Schematic representation of full length *Tb*REL1 and the three truncation mutants. (1) *Tb*REL1 full length, showing the five motifs (I–V) common to all members of the nucleotidyl transferase family, along with R372 and the DALKD motif (2) *Tb*REL1-N term, containing the five signature motifs critical for catalysis. (3) *Tb*REL1-R372, containing the five N-terminal motifs and R372 (4) *Tb*REL1-DALKD, containing the DALKD motif and lacking the final 59 amino acids. (B). Recombinant *Tb*REL1 truncation mutants were expressed, precipitated using His-tag isolation beads and separated by 10% SDS-PAGE. (C) Co-precipitation of KREPA2 with full-length *Tb*REL1 and truncation mutants. A KREPA2 only control was run as a negative control to check for background binding of KREPA2 to the His-tag isolation beads. All truncation mutants were unable to pull down KREPA2.

Three *Tb*REL1 truncation mutants were constructed lacking different amounts of the C-terminus, while retaining the N-terminal (32 kDa) domain. The N-terminal region contains the adenylylation domain, including the five signature motifs (I-V) critical for catalysis and common to all members of the nucleotidyl transferase family (Fig. 3A). *Tb*REL1-R372 (38 kDa), similar to the previously reported construct [22], includes the motifs I-V along with an important arginine 372 that is conserved in *Tb*REL1 and related ligases and is important for catalysis. *Tb*REL1-DALKD (41 kDa) contains the entire N-terminal region and part of the C-terminal region, including the DALKD motif, which is conserved among kinetoplastids. This truncation mutant lacks the final 59 C-terminal amino acids. All truncation mutants were labeled with [^35^S]-methionine (Fig. 3B).

### Role of the TbREL1 C-terminus in the KREPA2 interaction

All *Tb*REL1 proteins were precipitated in the absence (Fig. 3B) and presence of KREPA2 (Fig. 3C). The three different *Tb*REL1 truncation mutants were unable to precipitate KREPA2; in contrast, full-length *Tb*REL1 precipitates KREPA2 in a robust manner (Fig. 3C). A KREPA2-only control shows low non-specific binding. These data suggest that the *Tb*REL1 region downstream of the DALKD motif is important for interaction with KREPA2, however, the possibility that these truncations bring about structural perturbations forming non-functional *Tb*REL1 cannot be ruled out.

### Identification of specific TbREL1 residues that regulate the interaction with KREPA2

**Fig 4:**
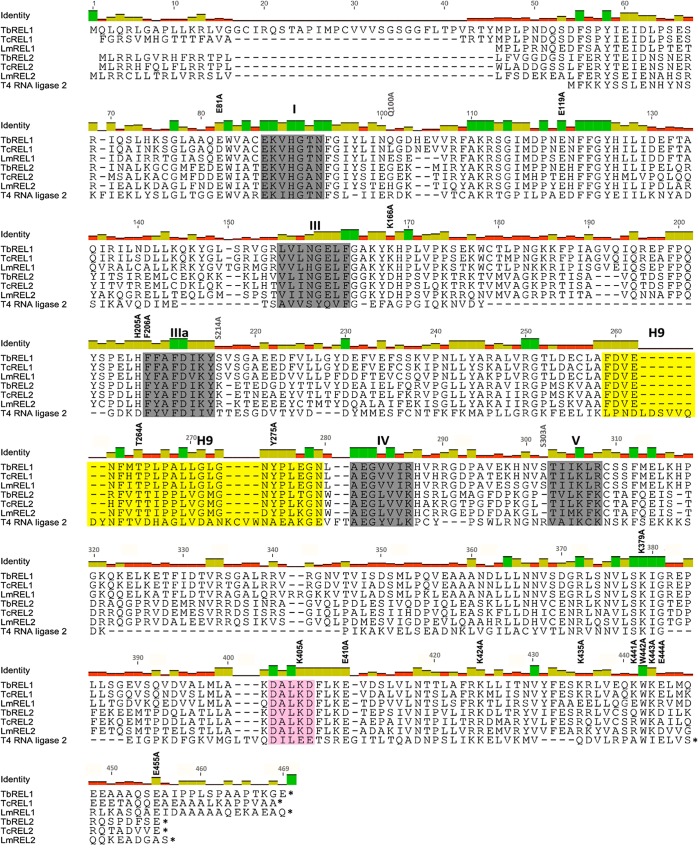
Multiple sequence alignment between kinetoplastid RNA editing ligases and T4 RNA Ligase 2. The amino acid sequences of *Tb*REL1 and *Tb*REL2 from *Trypanosoma brucei* were aligned with sequences from the corresponding ligases from *Trypanosoma cruzi*, *Leishmania major*, and T4 bacteriophage. Nucleotidyl transferase motifs I–V are shown in shaded boxes. The hydrophobic loop H9 and DALKD motif are highlighted in yellow and pink. The 17 point mutations used in the present study are labeled above the corresponding wild-type residues in black, and the 3 control point mutations are labeled in grey.

Seventeen individual *Tb*REL1 alanine substitution point mutations were synthesized for studying their effects on KREPA2 interaction, and ultimately, on *Tb*REL1 enzymatic activity. These seventeen target residues were selected on the basis of: (1) being charged, conserved residues in kinetoplastid RNA editing ligases and T4 RNA ligase 2 [12]; (2) previous reports on point mutations affecting T4 RNA Ligase 2 activity [23, 24]; (3) computer predictions regarding the structure of N-terminal residues important for protein-protein interactions [13, 25, 26]; and (4) predictions made by interaction algorithms developed in our laboratory (Najafabadi, unpublished). The point mutations are located in the N- and C-terminal regions of *Tb*REL1 (positions shown in Fig. 4). Apart from these seventeen, three arbitrary point mutations (Q100A, S214A and S303A), specifically in close proximity to motif I, IIIa and V (Fig. 4), were made as controls for all experiments.

To assess potential effect of TbREL1 mutations on the conformation of the proteins we analyzed the labelled protein on native gels. All point mutants were labeled with [^35^S]-methionine and resolved on a 4–16% Native gel (S2 Fig.). The migration of all mutants were identical to the wild-type *Tb*REL1, suggesting no serious perturbation of protein structure due to mutation.

### Co-precipitation of TbREL1 point mutants with and without KREPA2

**Fig 5:**
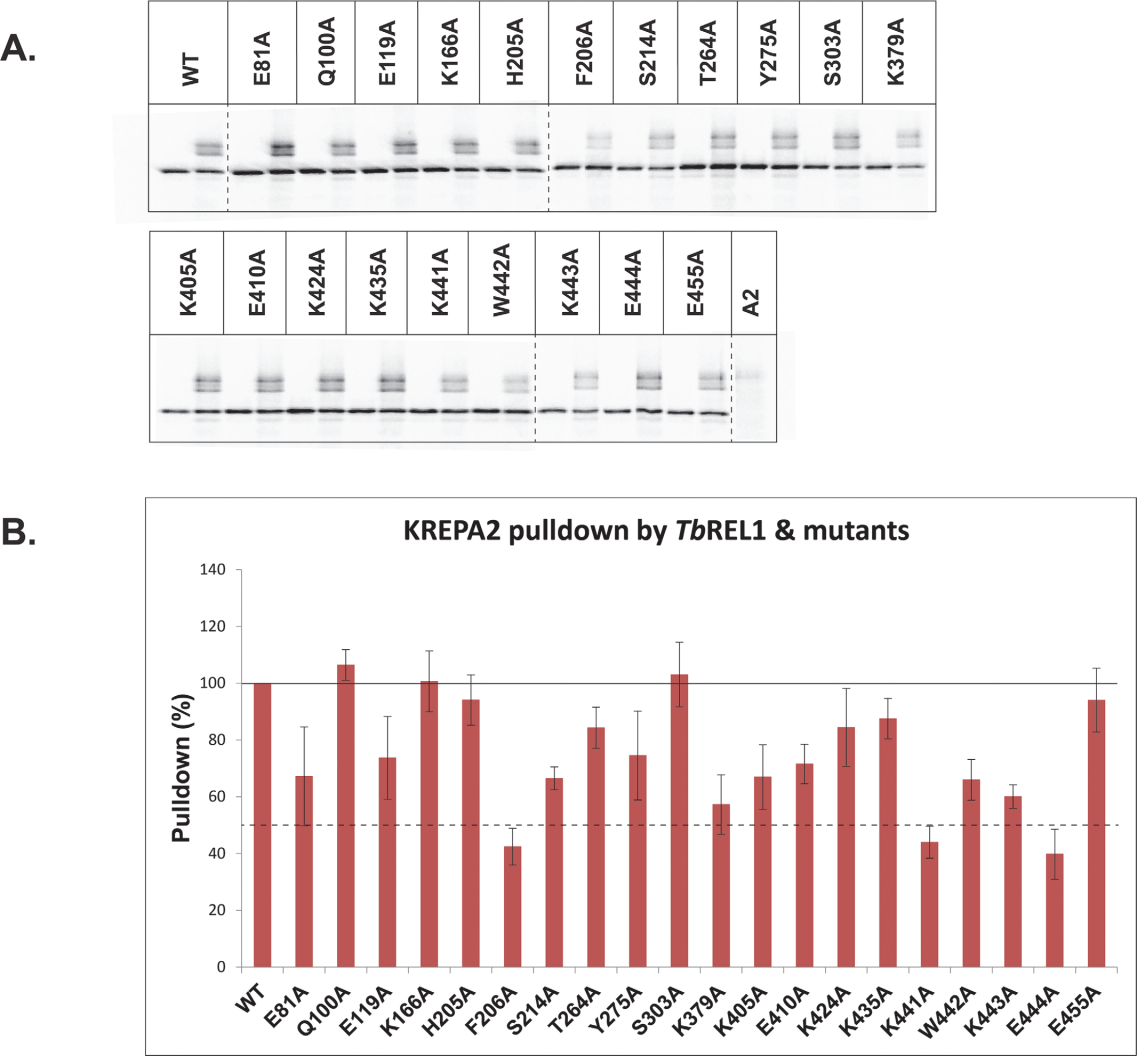
Interaction of *Tb*REL1WT and point mutants with KREPA2. (A) All mutants were expressed and precipitated in the absence and presence of KREAP2. KREPA2 was precipitated alone as a negative control to check for background binding to the his-tag isolation beads (last lane). (B) Graphical representation of the precipitation experiment. The amount of KREPA2 pulled down was first normalized with its own *Tb*REL1, after correcting for the background binding of KREPA2. Finally, all pull-down values were normalized to the amount of KREPA2 pulled down by *Tb*REL1 WT (100%). The amount of KREPA2 pulled down by *Tb*REL1 F206A, K441A, and E444A were less than 50% of the amount pulled down by the *Tb*REL1 WT. While the X-axis represents *Tb*REL1 WT and the different point mutants, the Y-axis represents relative pulldown (%). The error bars represent standard deviation between triplicate samples.

All *Tb*REL1 point mutations were precipitated in the absence and presence of KREPA2 (Fig. 5). The *Tb*REL1 point mutants precipitate KREPA2 with varying degrees of efficiency, in which three point mutants could do so with less than 50% capability. Of these, F206 is a part of motif IIIa and is highly conserved between T4 RNA Ligase 2 and all kinetoplastid RNA editing ligases. K441 and E444 are located in the extreme C-terminus, downstream of the *Tb*REL1 DALKD truncation point. Both residues have a charge conservation among related proteins (Fig. 4). A KREPA2-only control shows low non-specific binding to the magnetic beads (Fig. 5).

### Adenylylation of TbREL1 point mutants with and without KREPA2

**Fig 6:**
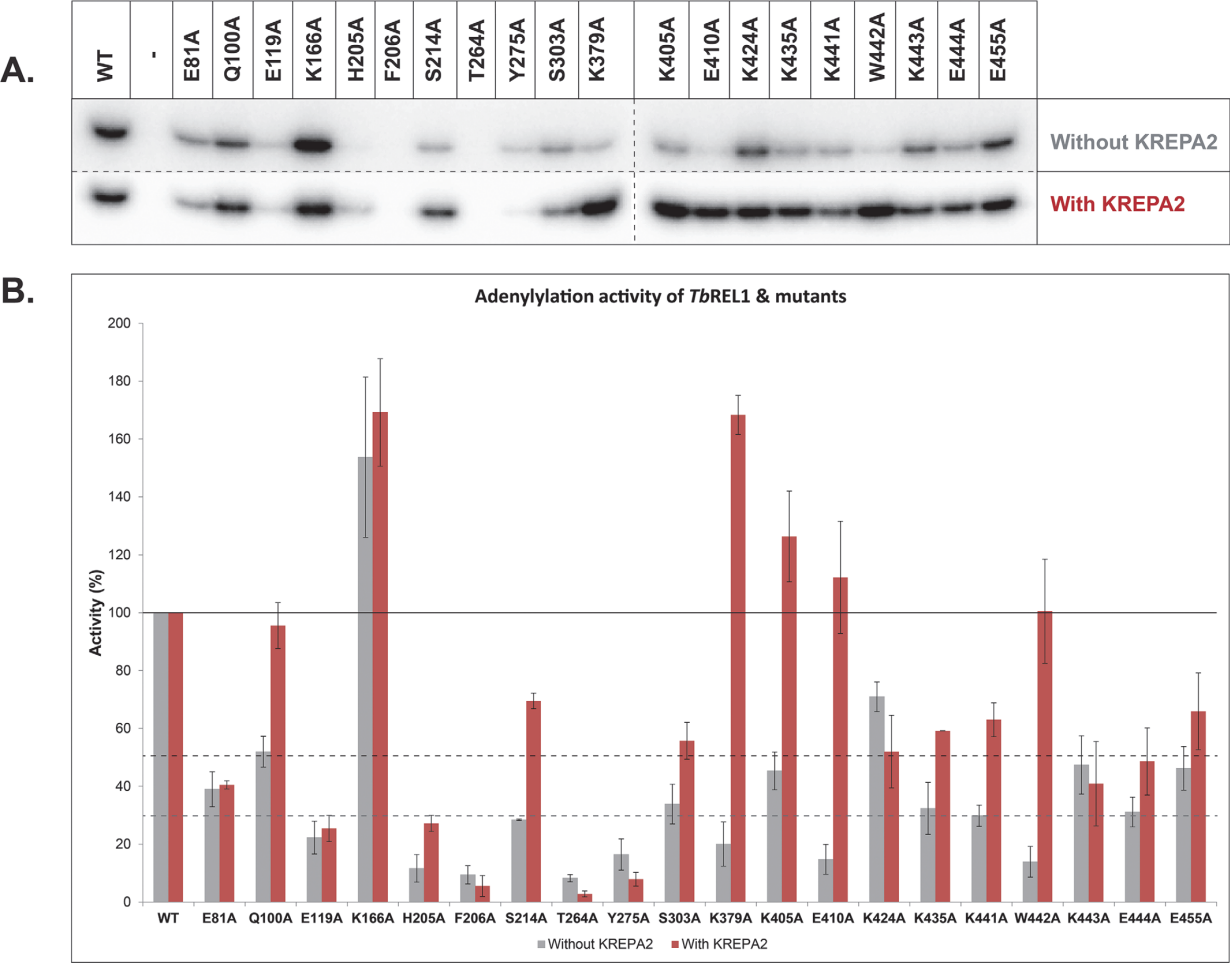
Adenylylation of *Tb*REL1 WT and point mutants in the absence and presence of KREPA2. (A) Adenylylation gel images of *Tb*REL1 WT and point mutants in the absence (top) and presence (bottom) of KREPA2. The intensity of the gel above was increased so that *Tb*REL1 WT adenylylated band intensities matched in both gels (with and without KREPA2), for the purpose of normalization. This gives a better visual perspective on the effect of each mutation with respect to the WT. (B) Graphical representation of adenylylation experiment. The intensity of each mutant in the top gel was normalized to its WT control, and the intensity of each mutant in the bottom gel was normalized to its WT + KREPA2 control. While the X-axis represents *Tb*REL1 WT and the different point mutants, the Y-axis represents relative adenylylation activity (%). The error bars represent standard deviation between triplicate samples.

We tested the ability of KREPA2 to stimulate the adenylylation activity of all *Tb*REL1 point mutants. Although all point mutations (including the control mutations and excluding K166A) affect the basal adenylylation capacity of the protein, with majority ranging at 30% efficiency, the result of this experiment (Fig. 6) enabled us to divide the *Tb*REL1 molecule into two main areas of interest: the N-terminal and C-terminal regions.

The low basal adenylylation of all point mutations in the N-terminus (Fig. 6; E81A, E119A, H205A, F206A, T264A, and Y275A) are either not rescued by KREPA2 or the enhancement by KREPA2 is too low to be considered significant. The relative catalytic activity of these mutant proteins was severely impaired compared to the control mutants, Q100A, S214A and S303A. Importantly, one of the more considerably affected mutations, F206A (Fig. 6), leads to a loss of KREPA2 binding, as well (Fig. 5). Furthermore, addition of KREPA2 does not rescue the reduced activity of this point mutation, suggesting that F206 may be crucial for *Tb*REL1 self-adenylylation and interaction with KREPA2. Residues T264A and Y275A (Fig. 6) are located in an exposed hydrophobic loop, conversed between kinetoplastid RNA editing ligases, which have already been implicated in mediating protein-protein interaction [13].

Contrary to the mutations in the N-terminus, the point mutations in C-terminus did not have such dramatic effects on adenylylation activity. Mutations K379A, K405A, E410A, K424A, K435A, K441A, W442A, K443A, E444A, and E455A (Fig. 6) are of interest because they fall within a region previously implicated in mediating interaction with KREPA2 [27]. Taking this into account, binding by KREPA2 completely ‘rescued’ four point mutations (K379A, E405A, E410A and W442A) defective in adenylylation activity, and displayed partial ‘rescue’ or no effect (no more enhancement than what KREPA2 has on *Tb*REL1 WT) on the remaining mutations (K424A, K435A, K441A, K443A, E444A and E455A). From the latter group of residues, K443 is conserved only among kinetoplastids, and K424 and E455 are partially conserved among various ligases. Except for K379, E405, E410 and W442, mutations at all other residues in the C-terminus region seem to have an effect on *Tb*REL1 adenylylation activity; even though those mutations imitate adenylylation levels similar to that of the control mutations (at Q100, S214 and S303), we cannot rule out the possibility that these C-termini mutations are in fact affecting adenylylation, and possibly KREPA2 interaction.

### Ligation activity of TbREL1 point mutants with and without KREPA2

**Fig 7:**
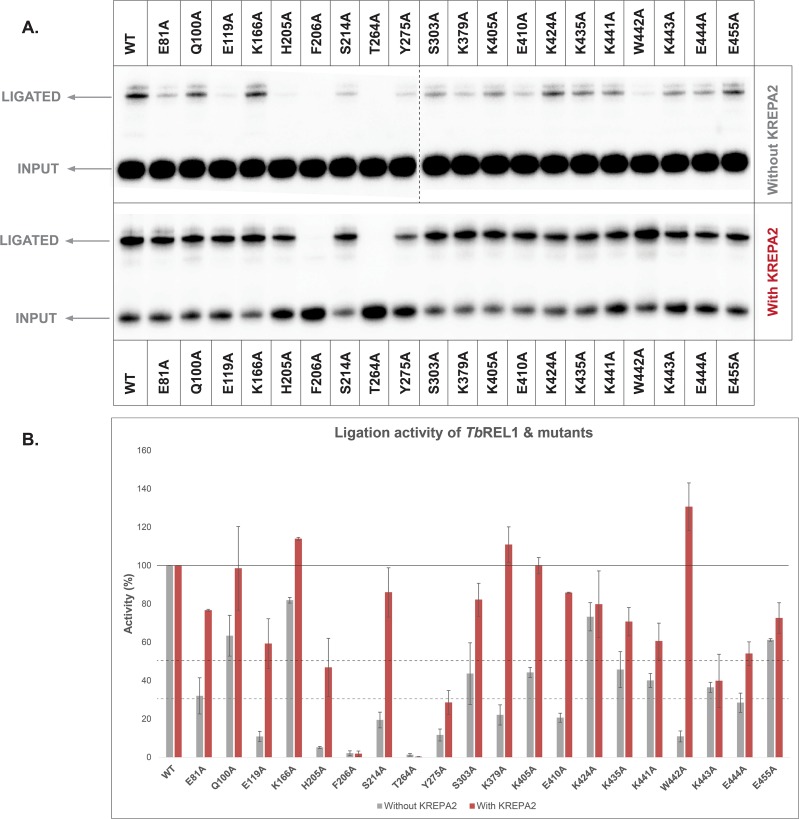
Ligation efficiency of *Tb*REL1 WT and point mutants in the absence and presence of KREPA2. (A) Ligation gel images of *Tb*REL1 WT and point mutants in the absence (top) and presence (bottom) of KREPA2. The intensity of the gel above was increased so that *Tb*REL1 WT ligation efficiencies could be visually perceived. The faint band seen above the ligated product is an artifact achieved from ligation occurring between 5’lig (16 bases) and glig (36 bases), instead of 3’lig (34 bases). (B) Graphical representation of ligation experiment. The intensity of each mutant in the top gel was normalized to its WT control, and the intensity of each mutant in the bottom gel was normalized to its WT + KREPA2 control. While the X-axis represents *Tb*REL1 WT and the different point mutants, the Y-axis represents relative ligation activity (%). The error bars represent standard deviation between triplicate samples.

All *Tb*REL1 point mutants were assayed for ligation activity in the presence and absence of KREPA2. Point mutations in the N-terminal region of *Tb*REL1 (E81A, E119A, K166A, H205A, F206A, T264A, and Y275A) showed a different pattern of ligation activity as compared to their adenylylation activity (Fig. 7). While ligation activity was enhanced considerably (enhancement is greater in fold change compared to WT *Tb*REL1) for E81A, E119A and H205A, the other point mutations, F206A, T264A, and Y275A, displayed defective ligation activity, similar to their adenlylylation capability, with exception of Y275A that showed a slight recovery in ligation in presence of KREPA2. Since a mutation at F206 affects KREPA2 pull-down, and KREPA2-stimulated ligase activities, it is possible that this residue plays a vital role in the KREPA2 interaction. Residues T264 and Y275 may also have roles in KREPA2 stimulation of *Tb*REL1.

Point mutants located within the *Tb*REL1 C-terminal region (K379A, K405A, E410A, K424A, K435A, K441A, W442A, K443A, E444A, and E455A) also showed robust ligation activity as seen for their adenylylation activity (Fig. 7). Mutants K379A, K405A, E410A and W442A showed reduced ligation activity that could be rescued to wild-type levels by the addition of KREPA2. While the point mutants, K441A, K443A, and E444A had slightly recovered upon KREPA2 interaction, K424A, K435A, and E455A showed a modest gain of activity by KREPA2 at the same level as for the WT. However, their effect on the catalytic activity of *Tb*REL1 was similar to the control mutations, Q100A, S214A and S303A. These data suggest that residues K441, K443, and 444, in the C-terminus play a role in KREPA2 mediated enhancement of *Tb*REL1.

## Discussion

RNA editing is an essential mechanism required for the viability of both, insect and bloodstream form stages of *T*. *brucei*. The editosome is an important complex, but its mode of action is yet to be fully understood. This study represents one of the first steps in designing chemical inhibitors against the interaction of the core components of this multi-protein complex. Several components of the editosome have already been studied, including the *Tb*REL1 ligase [11, 28, 29]. Evidence suggests that the interaction of ligases with their interacting partners is important for their function, but further investigations are required to clarify the mechanism of editosome function.

In this study we examined the interaction of *Tb*REL1 ligase with KREPA2, and the effect of this interaction on *Tb*REL1 adenylylation and ligation activities. We then tried to map the region of *Tb*REL1 responsible for KREPA2 interaction by constructing truncation mutants. However, truncated proteins often suffer from misfolding, leading to a loss of activity that may bias results. Thus, we went one step further and produced a number of *Tb*REL1 point mutants in an attempt to identify individual residues that mediate the KREPA2 interaction.

Upon interaction, not only does KREPA2 enhance the ligation activity of *Tb*REL1, but also its adenylylation activity (Fig. 2). This is surprising because KREPA2 only provides the necessary OB-fold domain required for binding RNA (lacking in *Tb*REL1), while *Tb*REL1 contains the necessary nucleotidyl transferase domain; suggesting that KREPA2 does not involve self-adenylylation of *Tb*REL1. Canonical OB-fold containing proteins, such as ATP-dependent DNA ligase and mRNA capping enzymes contain an essential motif (motif VI; RxDK) in their OB-fold region [30]. This motif is required for the enzyme nucleotidylation. Based on data on Chlorella virus capping enzyme bound to GTP, the RxDK motif is involved in the proper orientation of the pyrophosphate leaving group for an in-line attack on the lysine nucleophile. Accordingly, it is possible to infer from this information that KREPA1 and KREPA2 stimulate the adenylylation activity of *Tb*REL2 and *Tb*REL1 via the undiscovered motif VI in their OB-fold region. Even though, motif VI (RxDK) cannot be readily detected in the sequences of KREPA1 or KREPA2, it has been shown that this motif can exist with insertions [31]. An alignment between KREPA1 and KREPA2 reveals a conserved region between the two proteins—RxNxK (data not shown) within their predicted OB-fold [27]. Alanine substitution-based point mutations or deletion-based studies in this predicted KREPA1 / KREPA2 motif VI will be required to confirm this hypothesis.

Three truncation mutants were constructed in order to map the *Tb*REL1 interaction domain. While each mutant contained the entire N-terminus (considered essential for catalytic activity), they varied with respect to how much of the C-terminus was retained. Co-precipitation results indicate that none of the truncation mutants can stably bind KREPA2 (Fig. 3), suggesting that approximately 59 residues in the extreme *Tb*REL1 C-terminus coordinate interaction with KREPA2. Even though data from protein truncation experiments can be misleading due to possible risk of misfolding, these truncation mutants also suggest that the extreme C-terminus is crucial for the stability of the protein itself.

**Fig 8:**
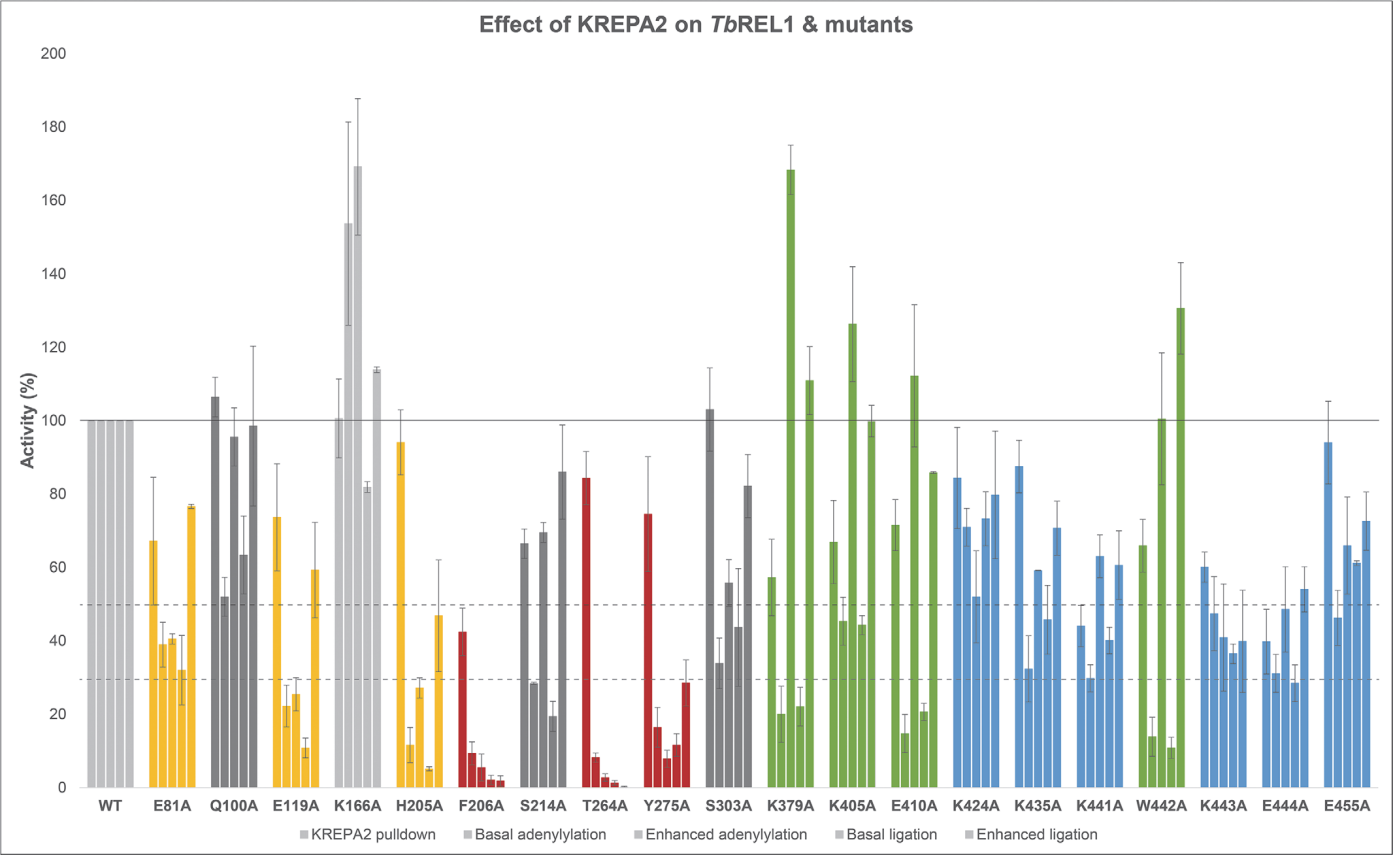
Graphical representation of the overall effect of KREPA2 on *Tb*REL1 WT and point mutants. The activity of *Tb*REL1 is segregated in two domains: N-terminal and C-terminal domains. Mutations in N-terminal domains lead to a considerable loss in adenylylation activity of *Tb*REL1, which are not rescued by the addition of KREPA2. While ligation activity of some of these point mutations are rescued (E81A, E119A and H205A; yellow bars), they remain affected for the others. Point mutations at F206, T264 and Y275 (shown in red bars) represent residues with severe effects on *Tb*REL1 enzymatic activity, with F206A having an effect on KREPA2 pull-down as well. While the overall activity of all *Tb*REL1 point mutants are affected in the C-terminal region, addition of KREPA2 completely rescues point mutations at K379, K405, E410 and W442 (shown in green bars), while having no effect or partially rescuing point mutations at the other residues, K424, K435, K441, K443, E444 and E455.

*Tb*REL1 contains a distinct N-terminal domain, known to take part and regulate adenylylation, and a much unannotated C-terminal domain, implicated to take part in interaction with KREPA2. Therefore, to further map the interaction site of KREPA2 on *Tb*REL1, seventeen alanine substitutions / point mutations were made at key residues in the ligase, and their effects on function were assessed. We tested these mutated proteins based on physical interaction with KREPA2, self-adenylylation (with and without KREPA2) and RNA ligation (with and without KREPA2) capabilities. All N-terminal mutants, except K166, showed a reduction in basal adenylylation and ligation activities (Fig. 8). This is not surprising as this region is known to be essential for the catalytic function of *Tb*REL1. The addition of KREPA2 did not rescue the loss of adenylylation in any of the N-terminal point mutants; although H205A showed some recovery in self-adenylylation in presence of KREPA2, the increase is not considerable. However, it is interesting to note that KREPA2 could rescue ligation activity, to a certain extent, of the N-terminal point mutants E81A, E119A and H205A. It is possible that these mutations affect only the adenylylation capability of the ligase, and the rescue seen in ligation activity may be as a result of an increase in turnover of the reaction due to the presence of KREPA2 and nicked RNA. Alternatively, it is possible that the ligase adenylylation step is bypassed in presence of KREPA2 and nicked RNA, since all other amino acids required for processing the ATP are active. Multiple point mutations at critical residues may be required to test this hypothesis.

Residue F206 is highly conserved among all kinetoplastid editing ligases and T4 RNA ligase 2; it is the only N-terminal residue for which a mutation disrupts KREPA2 binding for more than 50%, basal adenylylation and ligation, and also KREPA2 enhanced adenylylation and ligation (Fig. 8). The crystal structure of the *Tb*REL1 N-terminus [13] reveals that F206 is a buried residue; however, it may be exposed in the context of full-length *Tb*REL1 or become exposed upon interaction with KREPA2. Furthermore, this residue is part of the nucleotidyl transferase motif IIIa (Fig. 4), which has been predicted to play an important role in stabilizing the ATP interaction with TbREL1 [25]. Additionally, alanine substitution at F206 may disable the ligase from exposing this region, thereby, affecting KREPA2 binding in the process as well. This assumption is supported by the fact that alanine substitution of the adjacent residue, H205, does not lead to an effect as drastic as F206, leading to suggest that F206 is crucial for the overall activity of *Tb*REL1, by having a direct effect on KREPA2 binding, adenylylation and ligation activities. Nevertheless, the likelihood that F206A mutation disrupts protein tertiary structure, causing the above seen effects, cannot be ruled out. A more stringent method than Native-PAGE analysis, such as circular dichroism or X-ray crystallography may be required to establish the hypothesis.

The residues T264 and Y275 are part of a conserved, exposed hydrophobic helical—loop (helix 9; Fig. 4) that has been implicated in protein-protein interactions. Both residues are highly conserved among kinetoplastid editing ligases. T264 forms part of an interaction motif; an algorithm (developed by Najafabadi) predicts that this motif is involved in KREPA2 binding, while Y275 is also predicted to be involved in protein-protein interactions [25]. However, contrary to the predictions made, in the co-precipitation experiment (Fig. 5), KREPA2 interacts with these point mutants similar to the WT *Tb*REL1.

We propose an alternative hypothesis for these residues based on an interesting finding that this helical-loop is located right behind the adenylylation domain [13]; it is possible that these residues play a direct role in adenylylation. One of the above speculations made for residue F206 (part of motif IIIa) is to take part in adenylylation by exposing itself to access the ATP. It is likely that this loop is involved in the stabilization of that process, as both the loop and the exposed motif IIIa are mostly hydrophobic. This assumption is strengthened by our finding that the basal activities of T264A and Y275A point mutants are severely hampered, and are not rescued in presence of KREPA2, despite having a modest amount of interaction with KREPA2. This hypothesis implies a direct role for residues T264 and Y275 in adenylylation, possibly through stabilization of the motif IIIa (containing F206).

Mutations outside the essential N-terminal region do not have such dramatic effects on *Tb*REL1 catalytic activity. Most mutations, especially, K379A, E410A and W442A, show a significant decrease in basal adenylylation and ligation activities; however, upon binding of KREPA2 these activities are rescued up to wild-type *Tb*REL1 levels (indicating a greater fold increase in activity than that of the WT *Tb*REL1) (Fig. 8). Alanine substitution of K405A, a residue part of the DALKD motif, conserved in kinetoplastids and predicted to be important for catalysis, only shows a reduced basal level in activity and is completely rescued by KREPA2 similar to the mutations above. A mutation at the corresponding glutamic acid residue in T4 RNA ligase 2 showed no effect on adenylylation and ligation [24], mimicking the *Tb*REL1 + KREPA2 condition in our experiment (Fig. 8). This suggests that the DALKD motif may not be important for *Tb*REL1 and KREPA2 interaction, while we cannot rule out its role in the intergration with the editosome complex itself.

The KWKE motif downstream of the DALKD site seems to be required for interaction with KREPA2. Mutations affecting the two peripheral residues K441A and E444 disrupt KREPA2 binding (Fig. 8). All mutations (except W442A) in this region exhibit reduced overall activity, with and without KREPA2. Mutation in the corresponding tryptophan of T4 RNA ligase 2 has no effect on activity, similar to our W442A + KREPA2 condition. This information implies that certain residues are conserved between *Tb*REL1 and T4 RNA editing ligase 2, despite having poor sequence conservation between the two proteins in the C-terminus. However, this cannot rule out the possibility that the C-terminus of *Tb*REL1 has diverged from of the T4 RNA ligase 2 to incorporate other interactions required in the context of the editosome. This presumption is made from the finding that K441A and E444A disrupt KREPA2 interaction to a considerable extent. Moreover, point mutations at the extreme C-terminus leads to a lowered overall activity of the protein, with partial ‘rescue’ from KREPA2 interaction (Fig. 8), suggesting that the KWKE stretch of amino acids play a role in KREPA2 mediated enhancement of *Tb*REL1. Multiple point mutations at this stretch can be performed to test the hypothesis.

According to a proposed mechanism, KREPA2 binding to *Tb*REL1 causes a series of conformational changes required to coordinate sequential enzymatic steps [9]. If we picture the opening and closing of *Tb*REL1 to allow ATP entry, its conversion to AMP, and subsequent entry of the RNA substrate, then it is likely that different *Tb*REL1 residues are important for coordinating ATP binding and RNA-substrate binding. This may explain why many of the mutants have completely disrupted adenylylation activity, while their ligation activity can be rescued. On the whole, adenylylation activity seems to be more sensitive to point mutations, suggesting a more important role for KREPA2 in this stage of the reaction.

It has been shown that the KREPA2 binding to *Tb*REL1 is probably mediated by the C-terminus of the ligase [27]; our structural assays using *Tb*REL1 truncation mutants corroborate this finding. It is possible that the C-terminal residues K441, K443, and E444 initially coordinate KREPA2 binding, and a subsequent conformational change in *Tb*REL1 brings KREPA2 closer to the N-terminus, where it becomes closely associated with residues F206, T264, and Y 275, and together assists in adenylylation of the ligase.

## Conclusion

In the present study, we have shown that KREPA2 strongly stimulates the adenylylation and ligation activities of *Tb*REL1. We have mapped the region of contact with KREPA2 to the final C-terminal 59 amino acids of *Tb*REL1. Finally, we have identified several residues important for either the physical interaction with KREPA2 or the coordination of a possible conformational change brought about by KREPA2 binding and resulting in stimulation of *Tb*REL1 catalytic activity. Among them, the *Tb*REL1 N-terminal residues F206, T264, and Y275 are critical for catalysis as well as for interaction with KREPA2. The C-terminal residues K441, K443 and E444 part of the KWKE (442–444) sequence seem to coordinate the effects of KREPA2 on the overall activity of *Tb*REL1. Our studies indicate that multiple residues may coordinate KREPA2 stimulation of *Tb*REL1 adenylylation and ligation activities.

Further investigations using *Tb*REL1 double or triple mutants are required to verify the residues we have identified as possible points of contact for KREPA2 on *Tb*REL1. Single point mutations and multiple mutations on KREPA2 putative RxDK (motif VI) site may also benefit understanding the interaction between the two proteins.

## Supporting Information

S1 Fig Graphical representation of the intensities and protein amounts of the 50 KDa protein band form the 6x His protein ladder used in Fig. 1B. While the X-axis represents the amount of protein (ng), the Y-axis represents the intensity of the band obtained from the volume measurement tool in Quantity One.
(TIF)

S2 Fig Native-PAGE analysis of *Tb*REL1 WT and point mutants. All point mutants were run at the same voltage and identical times as the WT protein.
(TIF)
