# Supplementary figures and images for: Mutational Analysis of Trypanosoma brucei RNA Editing Ligase Reveals Regions Critical for Interaction with KREPA2
Source: PLoS One. 2015 Mar 19;10(3):e0120844. doi: 10.1371/journal.pone.0120844 (PMC4366279; doi:10.1371/journal.pone.0120844)

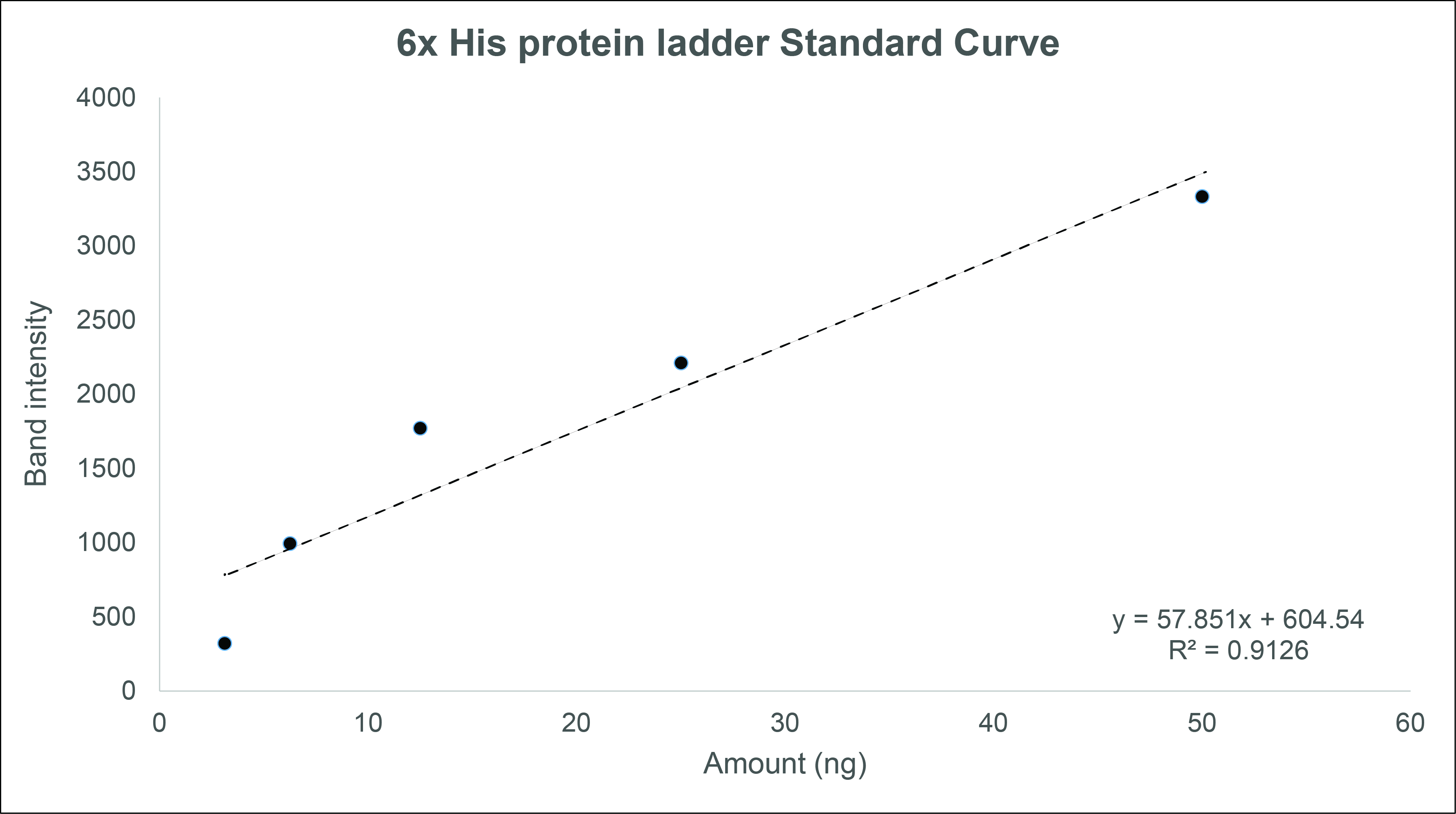

Supplement: S1 Fig — While the X-axis represents the amount of protein (ng), the Y-axis represents the intensity of the band obtained from the volume measurement tool in Quantity One. (TIF) [file pone.0120844.s001.tif]

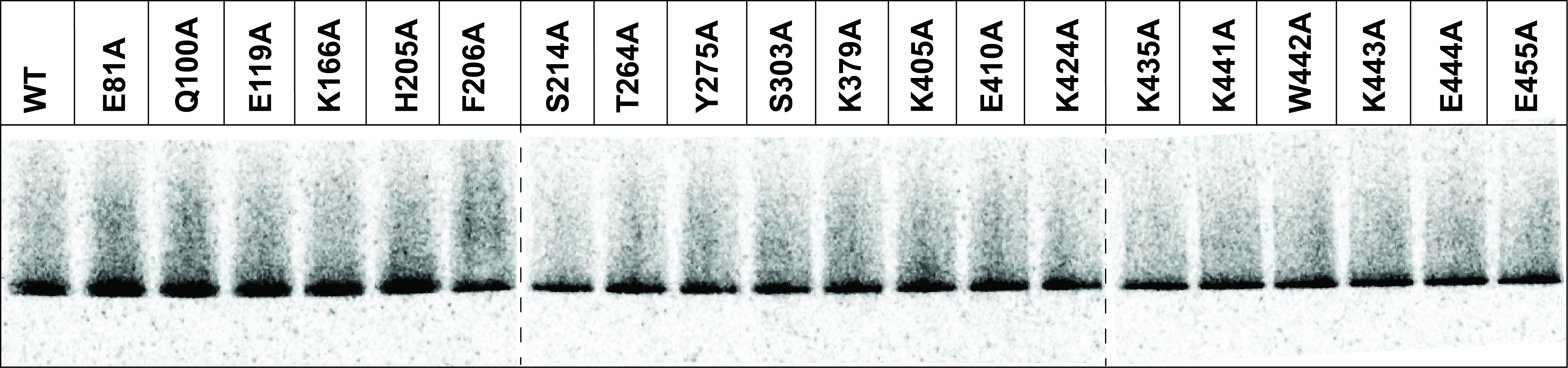

Supplement: S2 Fig — All point mutants were run at the same voltage and identical times as the WT protein. (TIF) [file pone.0120844.s002.tif]
